# A Rare Delayed Presentation of Uterine Rupture Following Vaginal Birth After Caesarean (VBAC): A Case Report and Literature Review

**DOI:** 10.7759/cureus.101906

**Published:** 2026-01-20

**Authors:** Victor Olagundoye, Obe John Ame, Najat Khenyab

**Affiliations:** 1 Department of Obstetrics and Gynecology, Women’s Wellness and Research Center, Doha, QAT

**Keywords:** caesarean section, ruptured uterus, scar rupture, vaginal birth after caesarean section, vbac

## Abstract

Uterine rupture is a rare and life-threatening obstetric complication that can lead to severe maternal and foetal morbidity and mortality. About 90% of uterine rupture occurs in a scarred uterus, often following a previous caesarean section or uterine surgery. Uterine rupture is the leading cause of maternal and neonatal morbidity in women attempting a trial of labour after a previous caesarean delivery. The rupture rate among women with a prior scar is approximately 0.5-0.7% and increases with the induction of labour and or augmentation. Rupture of an unscarred uterus is very rare, with an incidence of 0.4-0.6 per 10000, but is believed to be considerably higher in developing countries due to delays in recognising obstructed labour and providing early intervention. The sharp rise in caesarean section rates worldwide and the associated risks to maternal health have increased interest in vaginal birth after caesarean section (VBAC) as a safe and effective way to lower caesarean rates. A ruptured uterus presenting several days after a seemingly successful VBAC is very rare and can be challenging to diagnose, as symptoms and signs are non-specific and may be confused with routine puerperal symptoms, resulting in delayed diagnosis and treatment with long-term health consequences.

We report the case of a 35-year-old woman with three previous deliveries, who presented to the Emergency Department one week after a seemingly successful VBAC with complaints of mild vaginal bleeding, lower abdominal pain, and back pain resembling labour pain. Her first pregnancy resulted in a normal vaginal delivery followed by a caesarean section for maternal requests. She was considered a suitable candidate for VBAC due to her prior term normal delivery and, therefore, presumed to have an adequate pelvis given the size of the index foetus. Her labour onset was spontaneous, and her delivery and postpartum period were uncomplicated. She was discharged 24 hours after delivery. On examination, she was afebrile and hemodynamically stable. A pelvic ultrasound revealed a ruptured caesarean section scar surrounded by haematoma, which was confirmed by a computed tomography (CT) pelvis. Following discussion of the clinical problem and management options, she opted for surgical management, and she underwent laparotomy and repair of the ruptured scar. Her postoperative recovery was uneventful, and she was discharged within 24 hours.

The increased focus on VBAC as a safe and effective method to lower the rising caesarean section rate has led to more cases of ruptured uterus, usually during labour and delivery. Ruptured uterus presenting several days after what appears to be a successful VBAC is rare, but this is likely to change with the growing VBAC rate. A high level of suspicion is necessary to prevent delayed diagnosis and management, which could affect long-term health outcomes.

## Introduction

Uterine rupture is a rare and life-threatening obstetric complication with the potential for severe maternal and foetal morbidity and mortality. About 90% of uterine rupture occurs in a scarred uterus, often following a previous caesarean section or uterine surgery [1]. Uterine rupture is the leading cause of maternal and neonatal morbidity in women attempting a trial of labour after a previous caesarean delivery [1,2]. The rupture rate among women with a prior scar is approximately 0.5-0.7% and increases with the induction of labour and or augmentation [1]. Rupture of an unscarred uterus is very rare, with an incidence of 0.4-0.6 per 10000 [1]. The rising rate of caesarean sections worldwide and the associated increase in maternal complications from multiple caesarean deliveries have heightened interest in VBAC as a safe and effective alternative in carefully selected and motivated women [3].

Uterine rupture presenting several days after a seemingly successful vaginal birth following a caesarean section is extremely rare, with only a few cases reported in the literature. Diagnosis can be challenging because it lacks specific symptoms like those experienced during labour. As a result, symptoms may be easily mistaken for other postpartum conditions. A delay in diagnosis and treatment can lead to long-term health issues. An ultrasound or computed tomography (CT) of the pelvis is crucial for an accurate diagnosis.

Here, we present a rare case of uterine rupture at the site of a previous caesarean scar in a woman presenting to the emergency department seven days after an apparently uncomplicated, successful VBAC. This highlights the importance of remaining vigilant in women with a history of prior caesarean section undergoing VBAC, even after a seemingly successful attempt. Treatment may be conservative, involving prophylactic antibiotics depending on the extent of the rupture and the patient's symptoms. Definitive treatment involves surgical repair of the ruptured uterus with parenteral antibiotic cover, either via laparoscopy or laparotomy.

## Case presentation

A 35-year-old woman, para 3, presented to the Emergency Department one week after vaginal delivery following a caesarean section two years earlier. She reported mild vaginal bleeding, lower abdominal and back pain resembling labour pain. Her VBAC was uncomplicated, and she was discharged after 24 hours of delivery. Her first pregnancy resulted in a normal vaginal delivery, followed by an uncomplicated elective C-section at 39 weeks for maternal request after declining induction of labour for foetal growth restriction and abnormal Doppler findings.

On examination, she was afebrile. Her blood pressure was 118/61 mmHg, with a pulse rate of 103 beats per minute, and a respiratory rate of 19 breaths per minute. Her abdomen was soft with mild suprapubic tenderness, and the uterus was palpable at approximately 18 weeks' size and mildly tender. Vaginal examination showed a normal vagina and cervix with minimal bleeding through the cervical os. Blood investigations revealed a haemoglobin (Hb) level of 10.3 g/dL (post-delivery Hb was 12.0 g/dL), a white blood cell count of 11.7 × 10³/µL, C-reactive protein of 14.2 mg/L, and a platelet count of 471 × 10³/µL (post-delivery platelets were 223 × 10³/µL). Table 1 shows the laboratory parameters of the patient post-delivery, on admission, and at discharge.

**Table 1:** Key laboratory parameters of the patient post-delivery, upon admission, and at discharge.

Parameters	Normal range	Post-delivery	On admission	At discharge
Haemoglobin (g/dL)	12-15	12.0	10.3	10.1
White cell count (×10³/µL)	4-10	12.7	11.7	12.1
Red blood cell count (×10⁶/µL)	3.8-4.8	4.1	3.5	3.6
Haematocrit (Hct) %	36-46	34.5	30.2	30.1
Absolute neutrophil count (×10³/µL)	2-7	9.8	7.3	8.5
Platelets (×10³/µL)	150-410	198	471	466
C-reactive protein (mg/L)	0-5	-	14.2	-

**Figure 1:**
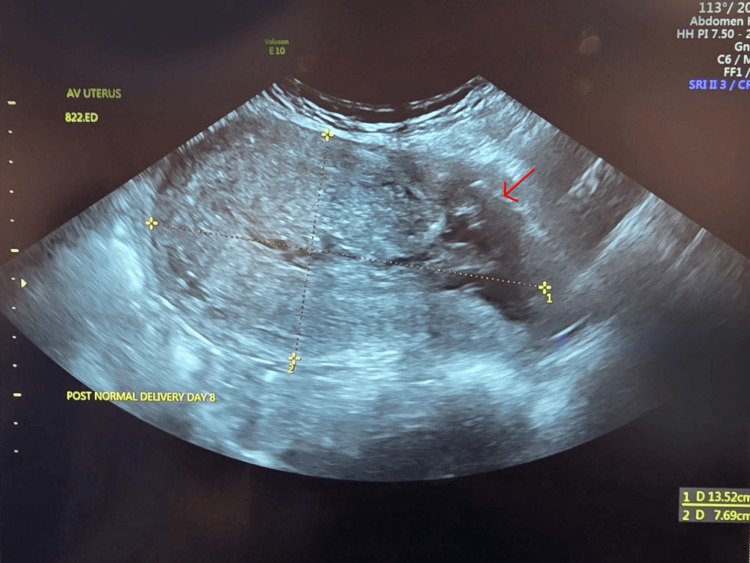
Pelvic ultrasound showing a large caesarean section scar defect (measures: 28 mm), with a large hematoma extending from the lower endometrial cavity through the open caesarean scar externally in between the uterus and urinary bladder.

**Figure 2:**
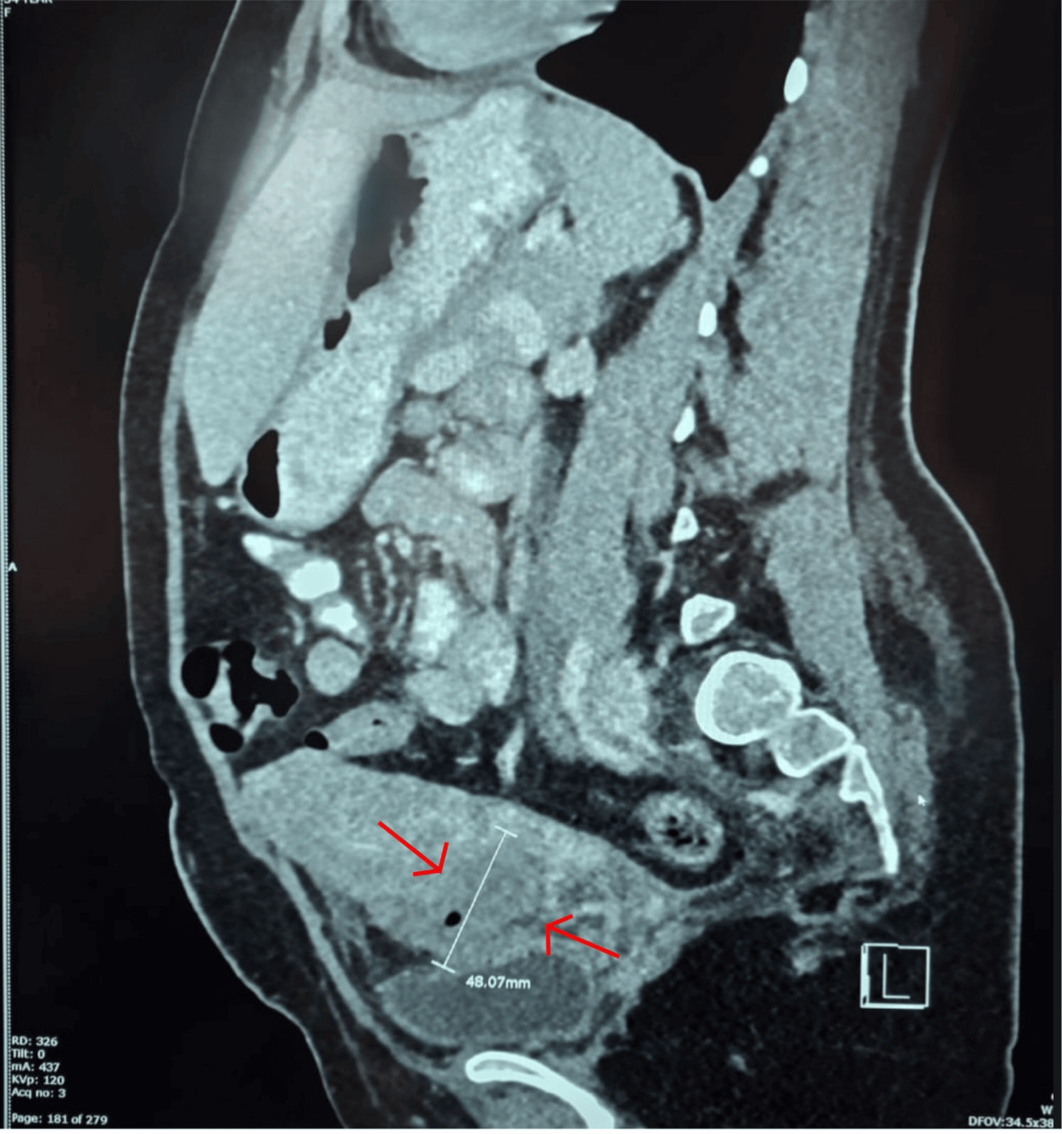
CT abdomen and pelvis showing a haematoma with air locules, extending from the lower endometrial cavity through the caesarean scar to the space between the uterus and urinary bladder.

Pelvic ultrasound showed a uterus measuring 136 × 92 × 77 mm, a caesarean scar defect of 28 mm, and a large haematoma measuring 61 × 61 × 56 mm with a volume of 109 mL extending from the lower endometrial cavity through the open caesarean scar externally, located between the uterus and bladder, as shown in Figure 1. A CT of the pelvis confirmed a ruptured uterine scar, as shown in Figure 2. A diagnosis of rupture caesarean section scar following VBAC was made.

**Figure 3:**
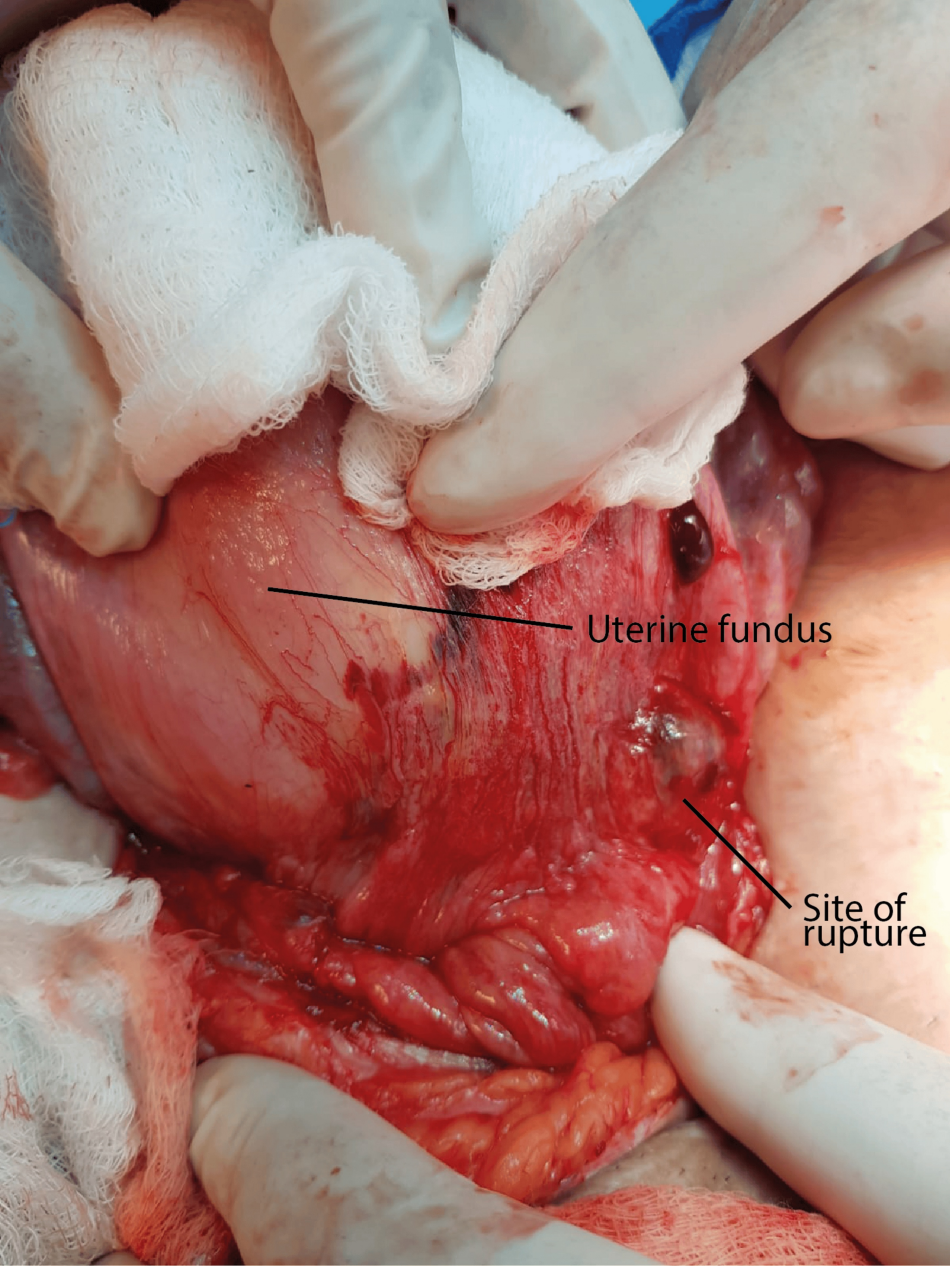
Findings at laparotomy showing ruptured caesarean section scar site partly covered with omentum.

The clinical problem and management options were discussed with the patient, and she chose surgical management. After obtaining informed consent, she was started on intravenous antibiotics and taken for laparotomy via the previous Pfannenstiel incision to repair the uterus. Findings at laparotomy included a sub-involuted uterus approximately 18 weeks in size, a small haemoperitoneum of about 50 mL, and a ruptured previous caesarean scar about 3 cm in length near the left uterine angle, as shown in Figure 3. The ruptured site was covered by a haematoma of approximately 6 × 6 cm, extending downward into the left broad ligament. The haematoma was evacuated, and the edges of the rupture scar were excised, followed by repair of the uterus in two layers using Vicryl sutures. The estimated blood loss was 100 mL. Her postoperative recovery was uneventful, and she was discharged after 24 hours. At the time of her discharge, she was advised to return to the Emergency Department if she experienced any problems, to monitor for abnormal bleeding or pain, and to keep a record of her menstrual periods. She was given an eight-week follow-up appointment by which time she would have had two menstrual cycles. At her eight-week review, she was well, with no complaints, and her menstrual cycle was regular with normal flow and no dysmenorrhoea. She was advised to avoid pregnancy for at least 12 months.

## Discussion

Uterine rupture is the pathological separation of all layers of the uterus during pregnancy or labour [4]. It is an obstetric emergency and remains the most feared complication for women undergoing a vaginal birth after a previous caesarean. This fear among medical professionals was emphasised by Edward Cragin’s statement in 1916 that “once a caesarean always a caesarean” [5]. This dictum has partly contributed to making repeat caesarean the leading factor in the rising caesarean section rate, with associated morbidity and financial costs [6]. Reconsideration of this paradigm has increased interest in VBAC. Uterine rupture remains the leading cause of maternal and neonatal morbidity in women attempting a trial of labour after a previous caesarean delivery, with a rupture rate of approximately 0.5-0.7% and increases with induction or augmentation of labour [1].

There has been a steady global increase in caesarean section rates over the last few decades, with the worldwide rate now at 21% [7]. Latin America and the Caribbean have some of the highest rates at 42.8%. The global average is projected to rise to over 30% by 2030 [8] and to more than 63% in Eastern Asia [8]. This trend has shifted emphasis towards VBAC as a safe and effective method to reduce the number of repeat caesarean sections in carefully selected and motivated women. VBAC is associated with decreased maternal morbidity and mortality during the index pregnancy and future deliveries. [2,3,9] The VBAC success rate ranges from 60-80% [6], with a higher rate of 80-90% among women with prior vaginal births.

The increasing number of women opting for VBAC has led to a rise in uterine ruptures and other related complications. Furthermore, a failed trial of labour after caesarean that results in a caesarean delivery is associated with greater morbidity than a planned caesarean [2]. These rising complications have caused some hospitals, especially in the USA, to stop offering trials of labour after a previous caesarean, leading to a decline in VBAC rate in the USA (TOLAC) [2,10]. The VBAC rate has also declined in the UK from 24.5% in 2017 to 14.2% in 2023 [9], possibly reflecting differences in counselling, consideration of obstetric history, and individual informed decision-making.

Our patient had one normal delivery followed by an elective caesarean section at 39 weeks on maternal choice as she declined induction of labour for foetal growth restriction and abnormal Doppler findings. A previous vaginal delivery, as in our case, or a prior successful VBAC remains the strongest predictor of successful VBAC, with a success rate of 85-90% [3]. Women considering a planned VBAC should be seen early in antenatal clinics to allow enough time to discuss delivery options. Dedicated antenatal clinics offer the additional benefits of providing structured and personalised care and counselling, along with emotional support that helps to alleviate fears of labour and uterine rupture, potentially increasing the rate of VBAC [11]. A previous history of uterine rupture, classical or T-incision, extensive trans-cavity myomectomy, or significant trans-fundal uterine surgery should be considered contraindications for VBAC due to the higher risk of uterine rupture [2].

Our patient attended a dedicated VBAC clinic at a tertiary hospital and was counselled about the risks and benefits of VBAC compared to an elective repeat caesarean section. Her previous operation note was reviewed, as per our practice, and she was considered suitable for VBAC. Our patient presented to the delivery suite in active labour and had continuous foetal heart rate (FHR) monitoring throughout labour. She progressed smoothly to a normal delivery. Her labour was not augmented, and she did not have an instrumental delivery. At no point was there any concern that she had a ruptured uterus. She was well post-delivery and had no complaints when she was discharged after 24 hours.

Most uterine ruptures occur during labour or immediately after delivery in women with a scarred uterus and can be catastrophic for both mother and baby. Therefore, it is advised that VBAC be carried out in a facility that is properly staffed, equipped with continuous electronic foetal monitoring, and capable of performing emergency caesarean section and advanced neonatal resuscitation. Early diagnosis of uterine rupture, combined with prompt surgical intervention and neonatal resuscitation, is vital in reducing morbidity and mortality. Zwart et al. [1] in a study involving 210 uterine ruptures, reported that 89.5% of uterine ruptures occurred during the intrapartum period, 9.5% before labour began, and 8.5% were diagnosed after delivery, with the highest incidence of uterine rupture occurring at 4-5 cm dilation.

There is no single pathognomonic clinical feature to diagnose uterine rupture in women undergoing VBAC; however, severe abdominal pain persisting between contractions, shoulder tip pain, abnormal vaginal bleeding or haematuria, abnormal cardiotocograph (CTG), and loss of uterine contractions should raise suspicion of rupture [1,3]. Vaginal palpation of the uterine scar after delivery to assess uterine scar integrity is not evidence-based and not supported by any of the colleges of obstetricians and gynaecologists, and should be discouraged. Women presenting days after an apparently successful VBAC lack these symptoms, making diagnosis very difficult, and their symptoms may be mistaken for normal puerperal effects. Our case involved a woman presenting seven days after delivery with non-specific symptoms of lower abdominal pain and mild bleeding. Murray et al [12]. described a patient who presented two days after an apparently successful VBAC with vomiting, lethargy, and abdominal pain, and was hemodynamically unstable, necessitating immediate laparotomy and uterine repair. In another case, Ali and Ali [13] reported a ruptured uterus presenting 23 days after VBAC with a growing abdominal mass and fever. They diagnosed puerperal sepsis and peritonitis secondary to uterine rupture and managed her with laparotomy and total abdominal hysterectomy. These cases highlight the varied clinical presentations and underscore the importance of vigilance in women undergoing VBAC, even after an apparently successful delivery, and emphasise the critical role of imaging for early diagnosis and management.

The decision to attempt a VBAC should be person-centred based on individual choice and risk assessment, with the aim of reducing risks and increasing the likelihood of success. This is important because the mode of delivery in the first pregnancy after a caesarean often influences future births. Factors associated with successful VBAC include a previous vaginal birth, spontaneous labour onset, and non-recurring indications for the prior caesarean, such as breech presentation [2,3]. Our patient had one vaginal birth followed by a caesarean for her second baby, making her a good candidate for a successful VBAC. Mercer et al. reported that the risk of uterine rupture decreases after the first successful VBAC [14]. They suggest this may be because multiparous women develop more effective uterine contractions during labour and have a lower risk of cephalopelvic disproportion (CPD). However, the reduced risk of rupture following a successful VBAC does not eliminate it entirely, and close monitoring during labour for signs of rupture or impending rupture remains essential.

The risk of caesarean scar rupture varies depending on the type of uterine scar. Women with a previous J or inverted T incision, a low vertical uterine incision, or inadvertent extensive uterine extension during the primary caesarean section have a two to three times higher risk of uterine rupture compared to women with a previous transverse incision [3,4]. Induction of labour with prostaglandin increases the risk of uterine rupture fourfold, while augmentation with syntocinon in the first or second stages of labour doubles the risk [3,15,16]. Since no single factor reliably predicts scar rupture, the decision whether to attempt VBAC or choose a repeat elective caesarean is a preference-sensitive choice based on patient values and preferences, and counselling should aim to support a shared decision on the mode of delivery, which should be documented in the medical record [2,3]. 

More than 95% of uterine ruptures occur intrapartum [1], with only a few isolated reported cases of delayed diagnosis, as seen in our case. A high degree of suspicion is necessary for diagnosis. Although the uterine rupture probably occurred during labour or delivery, it appeared to have been contained within the broad ligament, with minimal haemoperitoneum, which meant she did not exhibit the typical symptoms and signs of uterine rupture usually seen during labour or delivery. Diagnosing it can be challenging, as the woman's symptoms may be mistaken for normal puerperal symptoms, making it difficult for healthcare professionals to identify, leading to delayed diagnosis and treatment. Pelvic imaging is essential for diagnosis, and an ultrasound scan is usually sufficient. However, CT is crucial when the diagnosis is in doubt with an ultrasound scan. Treatment involves conservative management with antibiotics or surgical repair of the ruptured uterus, depending on the patient’s symptoms, extent of rupture, and patient choice in accordance with person-centred care. Surgery can be performed via laparoscopy or laparotomy, depending on the surgeon's preference and expertise. Following discussion of management options, our patient chose the surgical option, and she underwent surgical repair via laparotomy based on the surgeon's preferred approach. Delayed diagnosis and treatment can result in long-term health complications.

We do not utilise ultrasound assessment of the uterine scar to decide on the suitability of our patient to undergo VBAC. The usefulness of ultrasound measurement of lower uterine segment thickness in predicting uterine rupture among women with previous caesarean delivery has been examined in various studies [17]. The overwhelming conclusion is that the thinner the lower uterine segment scar, the greater the risk of rupture during VBAC [6,17]. However, there is no consensus, particularly regarding the timing of scans or what constitutes a safe lower uterine segment thickness. Additionally, several confounding factors that influence VBAC success cannot be explained solely by scar thickness.

## Conclusions

The rising caesarean section rate has heightened focus on VBAC to reduce the incidence of repeat caesarean deliveries. VBAC carries the risk of uterine rupture, typically during labour and delivery. A patient presenting with a ruptured uterus several days after delivery is very rare. With a renewed emphasis on VBAC, healthcare professionals are more likely to encounter cases where a seemingly successful VBAC later presents with a ruptured uterus. This case highlights the importance of treating VBAC cases as high risk with close monitoring, even after an apparently successful VBAC, and, when necessary, avoiding early discharge, to facilitate early diagnosis. It is important to highlight these cases and raise awareness so that healthcare professionals can remain vigilant, avoid delays in diagnosis, and achieve good outcomes.
